# Optimizing nitrogen application rate and plant density for improving cotton yield and nitrogen use efficiency in the North China Plain

**DOI:** 10.1371/journal.pone.0185550

**Published:** 2017-10-05

**Authors:** Pengcheng Li, Helin Dong, Cangsong Zheng, Miao Sun, Aizhong Liu, Guoping Wang, Shaodong Liu, Siping Zhang, Jing Chen, Yabing Li, Chaoyou Pang, Xinhua Zhao

**Affiliations:** Institute of Cotton Research of Chinese Academy of Agricultural Sciences, State Key Laboratory of Cotton Biology, Anyang, Henan, China; University of Delhi, INDIA

## Abstract

Plant population density (PPD) and nitrogen (N) application rate (NAR) are two controllable factors in cotton production. We conducted field experiments to investigate the effects of PPD, NAR and their interaction (PPD × NAR) on yield, N uptake and N use efficiency (NUE) of cotton using a split-plot design in the North China Plain during 2013 and 2014. The main plots were PPDs (plants m^−2^) of 3.00 (low), 5.25 (medium) and 7.50 (high) and the subplots were NARs of 0 (N-free), 112.5 (low), 225.0 (moderate) and 337.5 (high). During both 2013 and 2014, biological yield and N uptake of cotton increased significantly, but harvesting index decreased significantly with NAR and PPD increasing. With NAR increasing, internal nitrogen use efficiency(NUE) decreased significantly under three PPDs and agronomical NUE, physiologilal NUE, nitrogen recovery efficiency(NRE) and partial factor productivity from applied nitrogen (PFPN) also decreased significantly under high PPD between two years. Lint yield increment varied during different PPDs and years, but NAR enhancement showed less function under higher PPD than lower PPD in general. Taken together, moderate NAR under medium PPD combined higher lint yield with higher agronomic NUE, physiological NUE, and NRE, while low NAR with high PPD would achieve a comparable yield with superior NRE and PFPN and high NAR under high PPD and medium PPD produced higher biological yield but lower harvest index, lint yield and NUE compared to moderate NAR with medium PPD. Our overall results indicated that, in this region, increasing PPD and decreasing NAR properly would enhance both lint yield and NUE of cotton.

## Introduction

Planting population density (PPD) and nitrogen (N) application rate (NAR) are important controllable factors in cotton production. The PPD has been shown to significantly affect both leaf morphology and canopy photosynthetic characteristics of cotton, and the optimal spatial distribution of both light and specific leaf area is key for efficient utilization of light and N in cotton canopies [1]. Under a certain range of PPD, as PPD increases, plant height and leaf area index (LAI) of cotton increases and the timing of peak LAI is delayed [2]. In addition, the number of fruit branches per plant, boll weight and lint percentage decreases along with the number of buds, flowers, and boll per cotton plant at different growth stages [3]. An appropriate PPD of cotton provides a good microecological environment and is beneficial for coordination contradiction between groups and individuals [4]. This ensures that the individuals develop healthily without senescence, that a certain number of plants is maintained at the same time, and coordinates the development of the number of plants, boll number, boll quality per unit area of cotton field, to achieve high yield [5].

Nitrogen nutrition is one of the most crucial facets of cotton production. In a certain range of NAR, yield of cotton has been shown to increase as NAR increases, but excessive NAR leads to excessive vegetative development and delayed maturity, and thus a decreased yield and N use efficiency (NUE)[6–9].

It has been reported that an excess of about 50 kg N ha^−1^ was applied to cotton fields in Australia and 15–25% of the N fertilizer inputs may be safely reduced without yield reduction [10]. Dong et al. also suggested that N fertilizer can be used at a moderately lower rate and more efficiently than in traditional methods. They also found a significant interaction between PPD and NAR on lint yield in two fields with varying fertility, and showed that increased PPD or NAR enhanced biological yield but reduced harvest index[5]. Zhang et al. indicated that optimum PPD of cotton depends on NAR, salinity level, and other agronomic practices in saline field[11]. In a medium fertility field of the North China Plain cotton area, conventional NAR for cotton is 270–300 kg N ha^−1^ with a conventional PPD of 5.25 plants m^−2^, and lint yield 1700 kg ha^−1^ [12]. However, to our knowledge, it remains unclear whether controlling PPD or NAR is the best practice for cotton production in the region and information is lacking on the interaction of PPD and NAR on N uptake and the NUE of cotton. It is necessary to investigate the effects of increased NAR and decreased PPD or decreased NAR and increased PPD on yield, N uptake and NUE of cotton. Therefore, our main objectives were to evaluate the effects of PPD, NAR and their interaction on N uptake and the NUE of cotton and to determine optimum PPD and NAR based on both yield and NUE.

## Materials and methods

### Field experiments

Experiments were carried out on an experimental farm at the Institute of Cotton Research of the Chinese Academy of Agricultural Sciences in Anyang, China (36°06′N, 114°21′E, 76 m above sea level). The top 20 cm of soil at the site was a clay loam with pH 8.3, organic matter 11.8 g kg^−1^, available N 65.5 mg kg^−1^, available P 15.4 mg kg^−1^ and available K 131.5 mg kg^−1^. The monthly rainfall, average air temperature and sunshine duration during the growing seasons (April–October 2013 and 2014) are presented in Table 1. We used CCRI 79, a high-yielding commercial transgenic cotton cultivar carrying both the *Bt* and *CpTI* genes in this experiment. A split-plot design with three replications was applied each year. The main plots were assigned PPD treatments (3.00, 5.25 and 7.5 plants m^−2^, hereafter referred to as low, medium and high PPD, respectively), while NAR of 0, 112.5, 225.0 and 337.5 kg N ha^−1^, hereafter referred to as control, low, moderate and high treatments, respectively, in the form of urea were assigned to the subplots. In this region 5.25 plants m^−2^ is the typical PPD and 270–300 kg N ha^−1^ is the typical NAR. Each subplot contained six rows of cotton, 9 m long with an inter-row spacing of 0.8 m.

**Table 1:** Monthly weather summary during the cotton growing season in 2013 and 2014 at Anyang, Henan, China.

Month	Average temperature (°C)		Precipitation (mm)		Sunshine duration (h)	
	2013	2014	2013	2014	2013	2014
April	14.1	16.0	6.1	45.6	184.3	200.8
May	21.6	22.8	57.1	31.8	149.9	273.3
June	25.8	26.0	76.5	37.2	200.2	217.1
July	27.3	26.9	226.0	129.4	177.4	223.4
August	28.0	25.1	36.6	46.1	271.0	215.3
September	21.5	20.6	16.0	156.1	150.3	122.0
October	15.4	16.7	10.6	4.3	185.1	158.8
Average/total	21.9	22.0	428.9	450.5	1318.4	1410.8

Half of each N rate was applied basally before planting, and the other half was topdressed at the early flowering stage. All plots received a basal rate of 120 kg ha^−1^ P_2_O_5_ as calcium superphosphate (42% P_2_O_5_) and 120 kg ha^−1^ K_2_O as potassium sulfate (51% K_2_O) based on local practice.

Sowing date and growth stage of cotton in 2 years were seen in Table 2. Using the manual hill-drop planting method, four to six seeds were dropped per hill into the prepared furrow at hill–hill distances within rows of 40.0, 23.8 and 16.7 cm for PPD of 3.00, 5.25 and 7.50 plants m^−2^, respectively. The seeds were quickly covered with moist soil from both sides of the furrow and then mulched with plastic film (0.008 mm) along the rows.

**Table 2:** Cotton growth period in 2013 and 2014.

Year	Sowing date	Emergence stage	Squaring stage	Initial flowering stage	Boll-opening stage
2013	04–23	05–02	06–10	07–08	08–23
2014	04–27	05–06	06–06	06–28	08–20

Seedlings were freed from mulching by cutting film above hills at full emergence, and thinned to 3.00, 5.25 and 7.50 plants m^−2^ by leaving one vigorous plant per hill at the two-leaf stage. Based on local practices, vegetative branches at nodes 3–6 on the main stems were manually removed around the peak squaring stage. The growth terminals on the main stems were also removed at the peak boll-setting stages (July 21 and 26 in 2013 and 2014, respectively). Throughout the growing season, plots were irrigated once in late June with an irrigation amount of 360 m^3^ ha^−1^. Other management practices, including pest and weed control, were conducted according to local agronomic practices.

### Sample collection and measurements

Data were collected for LAI, seed cotton yield, lint yield, yield components, biological yield, harvest index, agronomic NUE, physiological NUE, internal NUE, and nitrogen recovery efficiency (NRE).

In each year, three plants from the central four rows of each plot at the seedling, budding, flowering, boll-setting stage were manually uprooted for determining leaf area per plant using Li-3000 leaf area meter (Li-Cor, Lincoln, NE, USA), and LAI was calculated on a ground area basis. Plants from the central four rows of each plot were manually harvested three times (on 24 September, 21 October, and 19 November 2013; and on 23 September, 17 October, and 15 November 2014). Seedcotton (moisture ≤ 11%) was ginned on a 10-saw, hand-fed laboratory gin, and lint yield (kg/ha) as well as lint percentage (lint/seedcotton, w/w) was determined after ginning. Biological yield and yield components such as total number of bolls and boll weight were determined from 20 plants per plot randomly tagged at maturity. After harvesting of seedcotton, stalks (root, stem, branches, carpels and remnant leaves) from 20 tagged plants for each plot were removed from soil, air dried for 20–25 days and weighed, then the N content was determined using the Kjeldahl method. Biological yield was determined as seedcotton plus stalk yields. Harvest index was thus calculated as the ratio of seedcotton yield to biological yield. Pre-frost yield rate of cotton expressed as the percentage of the first two harvests to total harvests (by weight) was also determined.

### Calculation method of relevant parameters

There are four common types of NUE [13](Xu et al.,2012): agronomic NUE [14](Novoa et al.,1981), physiological NUE [15](Isfan et al., 1990), internal NUE [16](Witt et al.,1999), and NRE[17] (Jerzy et al.,2014). Different evaluation indexes evaluate NUE from different perspectives. Agronomic NUE evaluates N fertilizer investment benefit and it is linearly related to specific seedcotton and N fertilizer prices; physiological NUE comprehensively examines the efficiency of N uptake and use from the perspective of physiology; internal NUE reflects efficiency of formation of lint yield from N uptake; and NRE indicates how well cotton takes up the applied N fertilizer. It is suggested that profitability and N impact on the environment should both be considered at the same time for cotton production, so agronomic NUE and NRE are the most important NUE types for producers.

N uptake (kg ha^−1^) = Dry matter yield (kg ha^−1^) ×N %;
agronomic NUE = (Y_f_ − Y_0_)/F_appl_, where Y_f_ and Y_0_ refer to seedcotton yields (kg ha^−1^) in the treatment in which fertilizer N has been applied and was not applied, respectively; and F_appl_ is the amount of fertilizer N applied (kg N ha^−1^);
physiological NUE = (Y_f_ −Y_0_)/(TNU_f_−TNU_0_), where TNU_f_ is total N uptake of N-fertilized plots and TNU_0_ is total N uptake of zero-N plots;
internal NUE = lint yield/TNU_f_;
NRE = (TNU_f_ − TNU_0_)/NAR;
and partial factor productivity from applied N (PFPN) = Y_f_/ F_appl_.

### Data analysis

Stata 13.0 software (StataCorp LP, College Station, Texas, USA) was used for data processing. In the statistical analysis, PPD, NAR, and year were entered as fixed effects, while block (replicate) was entered as a random factor. The factor block was nested within year. Duncan’s multiple range tests were used to separate treatment means at the 5% level.

## Results

### Effects of PPD and NAR on yield and composition of cotton

#### Effects of PPD on biological yield, economic (seedcotton, lint) yield and harvest index

With the increase of PPD (under the same NAR), biological yield increased significantly, and number of bolls per m^2^, seedcotton yield and lint yield showed an increasing trend, while harvest index decreased significantly, and boll weight, pre-frost yield rate also showed a decreasing trend (Table 3). When PPD of 5.25 plants m^-2^ with an NAR of 337.5 kg N ha^-1^ were used as the standard treatment in 2013 and 2014 respectively, the average percentages for the two years showed that under N0 treatments (no N application), lint yield for medium PPD and for high PPD increased 7.4 percentage point and 8.1 percentage point than that for low PPD respectively, under low NAR, lint yield for medium PPD and for high PPD increased 10.9 percentage point and 9.7 percentage point than that for low PPD respectively, under the moderate NAR treatments, lint yield for medium PPD and for high PPD increased 15.0 percentage point and 7.3 percentage point than that for low PPD respectively, under the high NAR treatments, lint yield for medium PPD and for high PPD decreased 0.3 percentage point and 3.3 percentage point than that for low PPD respectively. These results indicated that PPD had the effect of increasing yield under different NAR treatments. Under the low NAR treatment, lint yield increased with increasing PPD, while under the N0(no N application), medium and high NAR treatments, the yield of the medium PPD treatment was greater than that of the high PPD treatment because of the decrease of harvest index under high PPD. The average lint yield of all treatments under the medium PPD treatment increased 10.4, 3.5 percentage point than that under low PPD and high PPD respectively. This was related to the greater number of bolls per unit area under medium and high PPD than that under low PPD, and a higher boll weight for low and medium PPD than for high PPD.

**Table 3:** Effects of PPD and NAR on biological, economic (seedcotton and lint) yield and harvest index in 2013 and 2014.

Treatment	Yield of seedcotton (kg ha^-1^)	Lint yield (kg ha^-1^)	Biological yield (kg ha^-1^)	Harvest index	Boll density (bolls m^–2^)	Boll weight (g)	Lint percentage (%)	Pre-frost yield rate (%)
PPD (plant m^-2^)								
3.00	3734.8/3800.9(89.8)^*^	1474/1681.4(88.5)	7898.1/7012.0(67.8)	0.477/0.547(133.0)	61.1/61.2(86.9)	6.12/6.20(103.3)	39.5/44.1(98.5)	83.3/79.1(111.0)
5.25	4046.7/4328.5(99.8)	1591.1/1938.4(98.9)	11037.7/9273.0(92.3)	0.368/0.468(108.6)	68.6/73.2(100.8)	5.90/5.91(99.0)	39.3/44.3(98.5)	79.8/75.9(106.4)
7.50	3966.7/4197.3(97.3)	1553.1/1850.9(95.4)	11540.4/9650.0(96.3)	0.355/0.440(103.2)	69.9/74.7(102.8)	5.68/5.62(94.7)	39.1/44.8(98.8)	76.7/69.3(99.8)
NAR (kg ha^-1^)								
0	3805.1/3885.7(91.7)	1490.6/1724.3(90.1)	8578.4/7498.0(73.1)	0.451/0.524(126.6)	62.7/67.1(92.3)	5.79/5.82(97.3)	39.2/44.5(98.6)	83.7/76.6(109.6)
112.5	3925.9/4132.3(96.1)	1542.7/1830.6(94.6)	9574.1/8288.0(81.2)	0.420/0.506(120.3)	63.5/70.0(94.9)	5.86/5.93(98.8)	39.3/44.4(98.6)	82.6/75.4(108.0)
225.0	3984/4258.3(98.2)	1575.4/1894.0(97.3)	10780.4/9008.0(89.9)	0.382/0.481(112.1)	63.2/71.3(95.6)	5.95/5.98(100.0)	39.5/44.3(98.7)	78.3/74.4(104.4)
337.5	3949.2/4159.2(96.7)	1548.7/1845.4(95.2)	11702/9784.0(97.7)	0.347/0.430(100.9)	63.9/70.4(95.5)	5.98/5.92(99.7)	39.2/44.4(98.5)	75.0/74.6(102.3)
PPD×NAR								
3.00×0	3633.5/3494.5(85.0)	1464.3/1566.7(85.0)	6997.3/6009.0(59.1)	0.519/0.582(143.0)	60.2/56.5(82.9)	6.04/6.18(102.4)	40.3/44.8(100.2)	86.1/80.3(113.7)
3.00×112.5	3721.4/3789.0(89.5)	1470/1657.7(87.7)	7352.6/6607.0(63.4)	0.506/0.574(140.3)	61.2/61.1(86.9)	6.08/6.20(102.9)	39.5/43.7(98.0)	86.5/80.6(114.2)
3.00×225.0	3763.6/3918.0(91.6)	1482.9/1722.0(89.8)	8071.8/7068.0(68.8)	0.466/0.554(132.5)	61.5/62.8(88.3)	6.12/6.24(103.6)	39.4/44.0(98.2)	81.9/77.8(109.2)
3.00×337.5	3820.7/4002.0(93.2)	1478.6/1779.2(91.3)	9170.7/8362.0(79.7)	0.417/0.479(116.4)	61.3/64.5(89.4)	6.23/6.20(104.2)	38.7/44.5(98.0)	78.6/77.6(106.8)
5.25×0	3903.9/4058.0(94.9)	1495.2/1799.2(92.4)	10106.1/8390.0(84.1)	0.386/0.484(113.0)	67.0/69.8(97.2)	5.83/5.81(97.6)	38.3/44.3(97.3)	82.7/78.5(110.2)
5.25×112.5	4013.0/4362.0(99.8)	1553/1964.1(98.6)	10767.8/9058.0(90.1)	0.373/0.482(111.0)	68.6/73.4(100.9)	5.85/5.94(98.8)	38.7/45.0(98.6)	81.5/76.6(108.1)
5.25×225.0	4193.6/4581.0(104.6)	1677.4/2062.0(104.8)	11295.6/9624.0(95.1)	0.371/0.476(110.0)	70.7/76.9(104.9)	5.93/5.96(99.7)	40.0/45.0(100.1)	80.3/77.1(107.6)
5.25×337.5	4076.3/4313.0(100.0)	1638.7/1928.3(100.0)	11981.4/10020(100.0)	0.340 /0.430(100.0)	68.2/72.5(100.0)	5.98/5.95(100.0)	40.2/44.7(100.0)	74.8/71.5(100.0)
7.50×0	3878/4104.5(95.2)	1512.4/1807.0(93.1)	8631.8/8086.0(76.0)	0.449/0.507(124.2)	70.3/74.8(103.1)	5.51/5.49(92.2)	39.0/44.0(97.8)	82.3/70.9(104.7)
7.50×112.5	4043.4/4246.0(98.8)	1605.2/1869.9(97.4)	10602/9201.0(90.0)	0.381/0.461(109.4)	71.6/75.4(104.5)	5.65/5.63(94.6)	39.7/44.0(98.6)	79.9/68.9(101.7)
7.50×225.0	3994.8/4276.0(98.6)	1566.0/1898.0(97.1)	12973.8/10333(105.9)	0.308/0.414(93.8)	68.8/74.3(101.7)	5.81/5.75(96.9)	39.2/44.4(98.5)	72.8/68.2(96.4)
7.50×337.5	3950.4/4162.5(96.7)	1528.8/1828.7(94.1)	13954/10969(113.3)	0.283/0.379(86.0)	68.9/74.1(101.6)	5.74/5.62(95.2)	38.7/43.9(97.3)	71.7/61.9(91.3)
Significance of factors(P>F)								
PPD	<0.0001	0.0001	0.0001	0.0042	0.0029	0.0001	0.5293	0.0035
NAR	<0.0001	<0.0001	<0.0001	<0.0001	0.0346	0.0019	0.0893	0.0723
PPD×NAR	0.0064	0.0001	0.0033	0.4124	0.0369	0.0457	0.157	0.4412

*Values at the left and right of slash were means in 2013 and 2014 respectively, and values in parentheses were averages in terms of percentages of control in 2013 and 2014, and PPD of 5.25 plants m^-2^ with an NAR of 337.5 kg N ha^-1^ were used as control in 2013 and 2014 respectively.

#### Effects of NAR on biological yield, economic (seedcotton, lint) yield and harvest index

The effects of NAR on biological yield, economic (seedcotton, lint) yield and harvest index in the two years were approximately the same. Under low PPD, with the increase of NAR, biological yield of cotton significantly increased, and number of bolls per m^2^, boll weight, seedcotton yield and lint yield increased. Furthermore, the harvest index and lint percentage showed a decreasing trend and pre-frost yield rate initially increased but later decreased.

When PPD of 5.25 plants m^-2^ with an NAR of 337.5 kg N ha^-1^ were used as the standard treatment in 2013 and 2014 respectively, Lint yield for low, medium and high NAR treatments increased 2.7, 4.8, 6.3 percentage point than that for N0(no N application), respectively. Under moderate PPD, the low, moderate and high NAR treatments increased lint yield by 6.2, 12.4 and 7.6 percentage point than for N0(no N application), respectively. Under the high PPD treatment, low, moderate and high NAR treatments increased lint yield by 4.3, 4.0 and 1.0 percentage point than that for N0(no N application), respectively. The average lint yield under moderate NAR was more than that under the low and high NAR treatments by 2.7 and 2.1 percentage point respectively.

The average percentages for the two years showed that under low PPD, lint yield for medium NAR and for high NAR increased 2.1 percentage point and 3.6 percentage point than for low NAR respectively. Under the moderate PPD treatments, lint yield for medium NAR and for high PPD increased 6.2 percentage point and 1.4 percentage point than for low NAR respectively. Under the high PPD treatments, lint yield for medium NAR and for high NAR increased 8.7 percentage point and 2.8 percentage point than for low NAR respectively. These results indicated that under the medium and high PPD treatment, lint yield under the moderate NAR treatment was higher than that under the high NAR treatment, and the result reversed under the low PPD treatment.

#### Effects of interaction of PPD and NAR on biological yield, economic (seedcotton, lint) yield and harvest index

In 2013, the biological yield, seed cotton yield and lint yield, and the boll weight were significantly affected by PPD, NAR and their interaction (PPD × NAR). Harvest index and pre-frost rate were affected by PPD, NAR, but not by PPD × NAR. Boll density was affected by PPD and PPD × NAR, but not by NAR. The high PPD treatment produced the greatest biological yield but the lowest harvest index. The biological yield under the high PPD treatment was 46.1% greater than that under the low PPD treatment, and the harvest index under the low PPD treatment was 33.3% greater than that under the high PPD treatment. The effect of NAR on biological yield and harvest index was similar to that of PPD. The high NAR treatment produced the greatest biological yield but the lowest harvest index. Biological yield was increased 22.2% at high NAR relative to low NAR, and the harvest index increased by 28.6% under the low NAR treatment compared with that under the high NAR treatment (Table 3). In 2014, biological yield, seed cotton yield, lint yield and harvest index were significantly affected by PPD, NAR and PPD × NAR. Boll density was affected by PPD, NAR, but not by PPD × NAR. Boll weight and pre-frost rate were affected by PPD, but not by NAR or PPD × NAR (Table 3).

Lint percentage over the two years was not affected by PPD, NAR or PPD × NAR. Analysis of component of economic yield showed that the effects PPD × NAR on economic yields (seedcotton, lint) were realized mainly by the product of boll weight and boll density. The products of boll weight and boll density under the medium PPD treatment with moderate NAR and the high PPD treatment with low NAR were relatively higher than those of the other treatments, indicating that these combinations of treatments generated the highest economic yields.

The biological yield of each treatment in 2014 was lower than that in 2013, but harvest index, boll density boll weight and lint percentage in 2014 were greater than those in 2013. This indicated that the economic yield was higher in 2014 than in 2013.

### Effects of PPD and NAR on LAI of cotton

**Fig 1:**
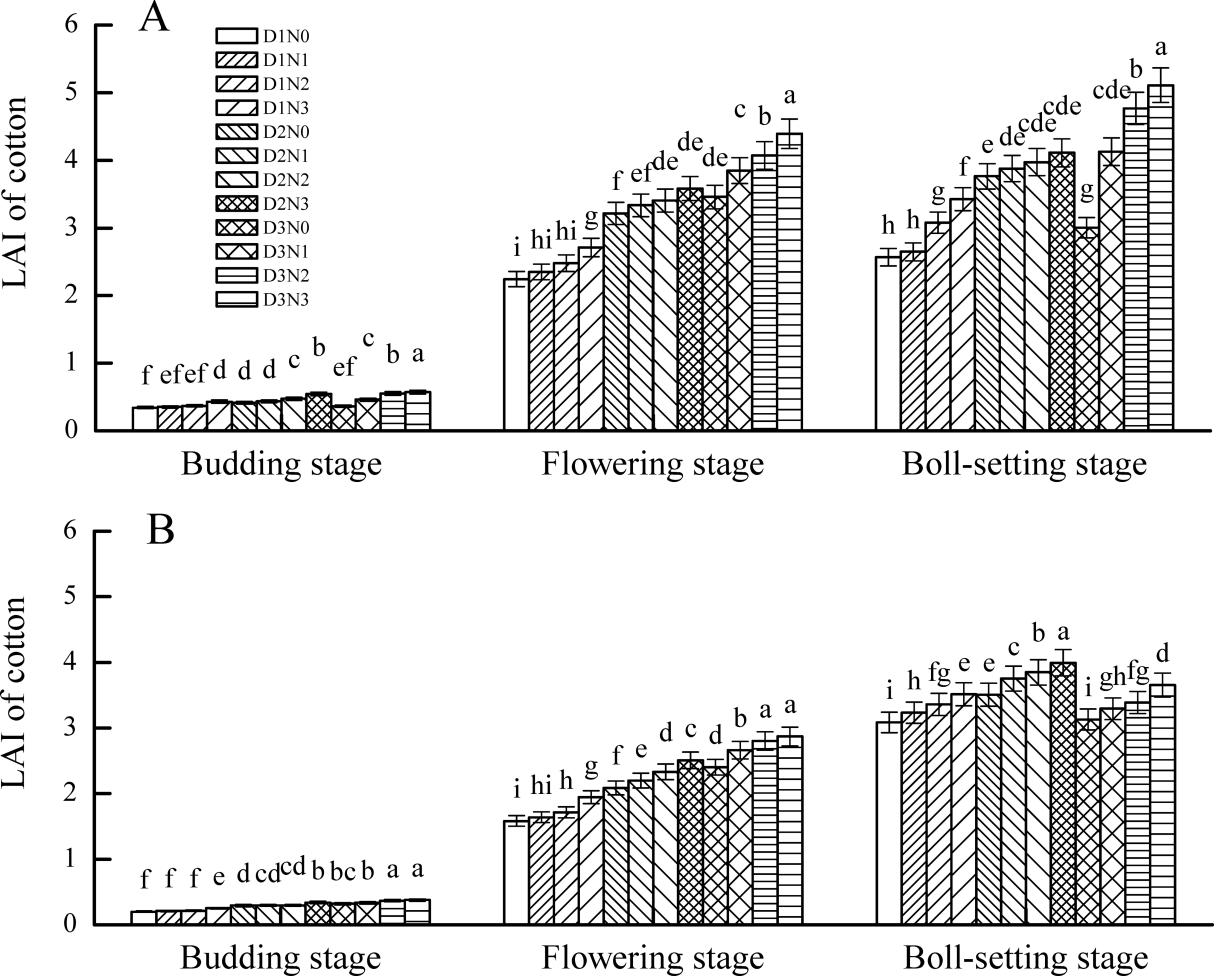
Leaf area index(LAI) of cotton at different growth periods in 2013(A) and 2014(B)Note: D1, D2, D3 indicate planting density at 3.00, 5.25, 7.50 plants m^−2^ respectively, and N0, N1, N2, N3, N4 indicate nitrogen application rate at 0, 112.5, 225.0, 337.5 kg ha^−1^ respectively. A, B indicate 2013 and 2014. Numbers at the same growth stage followed by the same small alphabet are not significantly different at the 5% level.

In 2013 (Fig 1), under the same PPD, the LAI of cotton at the squaring, flowering and boll-setting stages increased with the increase of NAR; however, NAR only affected LAI significantly under the high PPD treatment. Under the same NAR treatment, the LAI of cotton at squaring, flowering and boll-setting stage increased significantly with the increase of PPD. The LAI of cotton at different growth stages under high PPD with high NAR was significantly higher than that of the other treatments. These results indicate that there was a significant interaction between PPD and NAR on LAI of cotton at the squaring, flowering and boll-setting stages.

In 2014, under the same PPD, LAI of cotton at the squaring, flowering and boll-setting stages increased with the increase of NAR; however, NAR only affected LAI significantly under the medium PPD treatment. Under the same NAR, LAI of cotton at the squaring and flowering stages increased with the increase of PPD, but LAI at the boll-setting stage under the high PPD treatment was significantly lower than that under the low and medium PPD treatments. The LAI of cotton at the boll-setting stage under the treatment combination of medium PPD with high NAR was significantly higher than that of the other treatments.

The LAI of cotton for each treatment at the squaring, flowering and boll-setting stages in 2013 was higher than that in 2014. This result was the same for the biological yield of cotton in the two years, which was attributed to the much higher level of continuous rainfall in May, June and July in 2013 than in 2014 (Table 1), which promoted vegetative growth of cotton in 2013.

### Effects of PPD and NAR on N uptake and NUE of cotton

**Fig 2:**
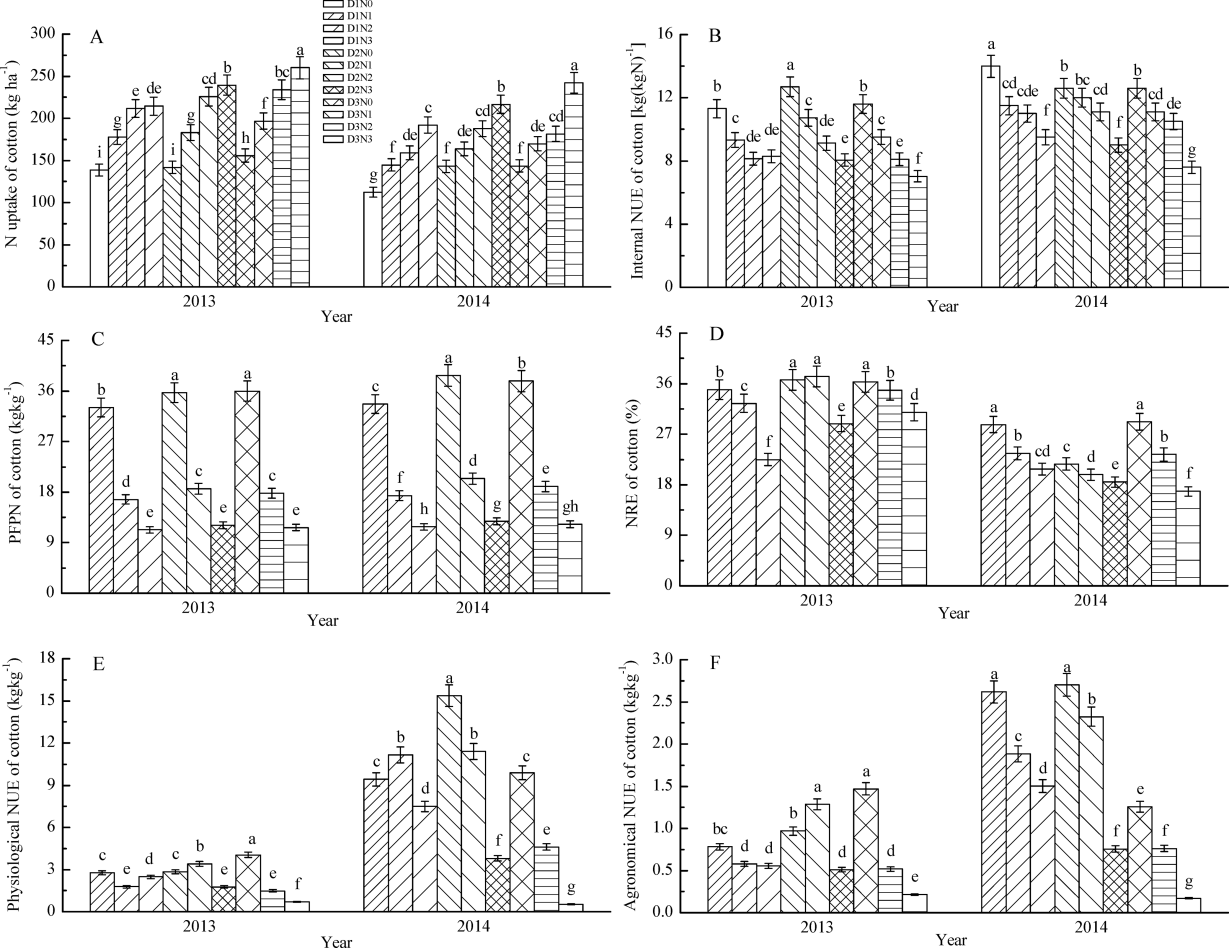
N uptake of cotton(A), internal nitrogen use efficiency of cotton(B), partial factor productivity from applied nitrogen of cotton(C),nitrogen recovery efficiency of cotton(D), physiological nitrogen use efficiency of cotton(E) and agronomic nitrogen use efficiency(F) of cotton in 2013 and 2014Note: D1, D2, D3 indicate planting density at 3.00, 5.25, 7.50 plants m^−2^ respectively, and N0, N1, N2, N3, N4 indicate nitrogen application rate at 0, 112.5, 225.0, 337.5 kg ha^−1^ respectively. Numbers for the same year followed by the same small alphabet are not significantly different at the 5% level.

Under the medium PPD (D2) and high PPD (D3) treatments, N uptake of cotton in 2013 and 2014 increased significantly with the increase of NAR, and the combination of high PPD and high NAR produced the greatest N uptake. Under the low PPD (D1) treatment, N uptake of the moderate and high NAR treatments differed significantly in 2014 but not significantly in 2013. Under the same NAR, N uptake increased with the increase of PPD; however, significant N uptake increase with the increase of PPD only occurred under the high NAR (N3) treatment (Fig 2).

In 2013, under the low and high PPD treatments, agronomic NUE of cotton decreased significantly with the increase of NAR, and under the medium PPD treatment, agronomic NUE under moderate NAR was significantly higher than that under low and high NAR. Under the low NAR treatment, agronomic NUE of cotton increased with the increase of PPD, while under the high NAR treatment, agronomic NUE decreased with the increase of PPD. Finally, under the moderate NAR treatment, agronomic NUE with medium PPD was more than that with low or high PPD (Fig 2).

In 2014, under the same PPD treatment, agronomic NUE of cotton decreased significantly with the increase of NAR. Under the same NAR, agronomic NUE of cotton under medium PPD was greater than that under low and high PPD. Agronomic NUE of cotton in 2014 was higher than that in 2013, which was related to the lower seedcotton yield of the control and higher seedcotton yield of the N input treatments in 2014 than in 2013 (Fig 2).

We found that there was a small increase in NRE of cotton under the moderate NAR treatment relative to the low NAR treatment under medium PPD in 2013 but this was not significant. In addition, NRE decreased with the increase of NAR under the same PPD in both years. Under low and moderate NAR in 2013, there were no significant differences in NRE between the different PPD treatments, while under the high NAR treatment, NRE increased with the increase of PPD. Under the low and moderate NAR treatments in 2014, NRE under moderate PPD was significantly lower than that under low and high PPD, while under the high NAR treatment, NRE decreased with the increase of PPD. The NRE of the same treatment in 2014 was lower than that in 2013, which may be related to the lower biological yield and N uptake of cotton in 2014 than in 2013 (Fig 2).

Under the same PPD, the internal NUE decreased with the increase of NAR in both years, and under the medium and high PPD treatments, NAR significantly affected the internal NUE of cotton in 2013. Under the control, low and moderate NAR treatments, internal NUE with medium PPD was higher than that with low and high PPD, and under the high NAR treatment, internal NUE with low PPD was higher than that with medium and high PPD in 2013.

Under low PPD, the low NAR treatment produced the highest physiological NUE in 2013 while moderate NAR produced the highest physiological NUE in 2014. Under high PPD in both years and medium PPD in 2014, physiological NUE decreased significantly with the increase of NAR. In contrast, under medium PPD in 2013, physiological NUE with the moderate NAR treatment was higher than that with low and high NAR. Under the low NAR treatment in 2013, physiological NUE with high PPD was superior to that with low and medium PPD. Under the low NAR treatment in 2014, physiological NUE with medium PPD was higher than corresponding values under low and high PPD. Under the moderate NAR treatment in the two years, physiological NUE of cotton with medium PPD was superior to that with low and high PPD, while under the high NAR treatment, physiological NUE decreased significantly with the increase of PPD. Physiological NUE under the same NAR and PPD treatments in 2014 was higher than that in 2013, which was attributed to the greater difference of seedcotton yield and lower difference of N uptake between N application treatments and the control in 2014 than in 2013 (Fig 2).

Under the same PPD treatment, PFPN of cotton decreased significantly with the increase of NAR. Under the low NAR treatment in 2013, PFPN of cotton increased with the increase of PPD, and there were no significant differences in PFPN among the different PPD treatments under the moderate and high NAR treatments in 2013. Under the low and moderate NAR treatments in 2014, PFPN under medium PPD was significantly higher than that under high and low PPD; however, there were no significant differences in the PFPN of cotton among the different PPD treatments under the high NAR treatment.

The results indicated that the low NAR with high PPD treatment produced much higher economic yield, agronomic NUE, PFPN, and physiological NUE, which was suitable for cotton production practice in the cotton areas although its yield was less than that of medium PPD with moderate NAR.

### Effective curve of PPD and NAR for seedcotton yield

According to the seedcotton yield results, two binary quadratic effect hyperboloid equations were established for seedcotton yield (Y), PPD (D) and NAR (N) in 2013 and 2014 as follows:

$$Y_{2013}=2520.65+472.50N+1.92D-38.71N^{2}-0.0031D^{2}-0.086ND,R^{2}=0.9356;$$

$$Y_{2014}=1590.71+819.61N+4.65D-65.07N^{2}-0.0068D^{2}-0.29AD,R^{2}=0.9687.$$

Two regression equations were significant at the 5% level. In 2013, the regression coefficient for N^2^ and ND were not significant but regression coefficients for D^2^, D and N were significant at the 1% level, while in 2014, the regression coefficient for ND was significant at the 5% level, and regression coefficients for N^2^, D^2^, N and D were significant at the 1% level. This shows that there was a significant effect of PPD on seedcotton yield, and there was an interaction of PPD and NAR on seedcotton yield; only under an appropriate combination of PPD and NAR was higher seedcotton yield achieved.

To further quantify a suitable PPD under different NAR treatments and provide a theoretical foundation for cotton production, seedcotton yield effect equations of PPD under different NAR in the two years were established, and the PPD for the highest seedcotton yield under different NAR treatments were calculated by derivation of the governing equation (Table 4). Seedcotton yield varied with an open downwards parabola as PPD changed, and seedcotton yield increased under a certain range of PPD. When PPD exceeded this range, seedcotton yield began to decline. Under the moderate and high NAR treatments, the PPD at the maximum seedcotton yield were 5.7, 5.6 plants m^−2^ in the two years, respectively, and the PPD at the maximum seedcotton yield was 6.6 plants m^−2^ in 2013 and 6.0 plants m^−2^ in 2014 under the low NAR treatment. The results showed that high PPD with a low NAR or medium PPD with moderate NAR or high NAR could obtain a much higher seedcotton yield.

**Table 4:** Seedcotton yield effect equation of PPD under different NAR treatments.

Year	NAR (kg ha^-1^)	Seed cotton yield effect equation under different NAR	Maximum of seedcotton yield(kg ha^-1^)	PPD at maximum seedcotton yield(plant m^-2^)
2013	0	*Y* = -29.264*D*^2^+ 361.61*D* + 2812.1	3 929.2	6.2
	112.5	*Y* = -25.798*D*^2^+ 342.43*D* + 2926.3	4 062.6	6.6
	225.0	*Y* = -62.104*D*^2^+ 703.47*D* + 2212.1	4 204.2	5.7
	337.5	*Y* = -37.679*D*^2^+ 424.45*D* + 2886.5	4 081.8	5.6
2014	0	*Y* = -51.062*D*^2^+671.7*D*+1938.9	4 147.9	6.6
	112.5	*Y* = -68.049*D*^2^+816.07*D*+1953.2	4 399.9	6.0
	225.0	*Y* = -95.605*D*^2^ +1083.4 *D* +1528.2	4 597.5	5.7
	337.5	*Y* = -45.58*D*^2^ +514.26 *D* +2886.5	4 337.0	5.6

## Discussion

### Effects of PPD and NAR on LAI of cotton

In the early 2000s, *Bt* cotton began to be planted in the Yellow River Cotton Area in China, and PPD of cotton in the area was about 4.5 plants m^−2^ considering both planting amount and seed costs. Chen et al.[18] indicated that a suitable LAI of cotton at the boll-setting stage was about 3.5, while Tan et al.[19] proposed that a suitable LAI range was 3.55–4.23, and a suitable LAI range of cotton in Hebei, Sichuan and Shandong province in China was 3.5–4.0[20]. In North Xinjiang Cotton Area of China, LAI of cotton at the boll-setting stage were 2.83, 3.25, 3.61 with 14.5, 19.5 and 29 plants m^−2^ respectively[21]. In this field experiment, the LAI of cotton at the boll-setting stage ranged from 3.51 to 4.11 under 5.25 plants m^−2^, in favor of the formation of cotton yield, while LAI of cotton under 3.00 plants m^−2^ ranged from 2.65 to 3.51, lower than the optimum LAI. Under 7.50 plants m^−2^, the LAI of cotton at the boll-setting stage at 225.0 or 337.5 kg N ha^−1^ in 2013 was 4.77 and 5.11, respectively, which was not conducive to a high yield, while LAI with 112.5 kg N ha^−1^ was 4.13, which was more favorable for a high cotton yield.

### Effects of PPD and NAR on cotton yield

The most suitable PPD for high output of cotton in different ecological regions varies because of the variability of climate, cultivated varieties and other agronomical practices. In downstream areas of the Yangtze River of China, the typical PPD for hybrid cotton variety was 3.0 plants m^−2^[22], while in North Xinjiang Cotton Area of China, the photosynthetic rate of cotton group at boll-setting stage with PPD 19.5 plants m^−2^ maintained a higher level, due to high light utilization efficiency, superior spatial distribution of leaf N allocation to the photosynthetic apparatus and photosynthetic use efficiency of photosynthetic N in leaves within the canopy[23], and the corresponding lint yield was also higher than that of cotton with PPD of 7.5, 31.5 plants m^−2^. In the South Xinjiang cotton area, lint yield with PPD of 18.0 plants m^−2^ was significantly higher than yields from fields under a PPD of 9.0, 13.5, 22.5, 27.0 plants m^−2^[24,25]. In the North China Plain, the difference in the lint yield of cotton between PPD of 5.1 and 8.7 plants m^−2^ was not significant, but lint yield of cotton with a PPD of 1.5 plants m^−2^ was significantly lower than the yields under a PPD of 5.1 and 8.7 plants m^−2^[26,27].

Lint yield of cotton is determined by combined action of yield component factors such as boll number of unit area, boll weight and lint percentage. In this field experiment, lint percentage was not affected by PPD and NAR, which may be because lint percentage is mainly determined by genetic factors[28]. Therefore, lint yield was primarily determined by the product of boll number and boll weight, and it was feasible to obtain a higher lint yield by better regulation of the relationship between boll number and boll weight[29,30]. Two variables (PPD and NAR) were introduced in this field experiment, which increased the complexity of regulation of boll number and boll weight. With the increase of PPD, boll number increased but boll weight decreased and only by increasing the product of the increased boll number and decreased boll weight could a higher lint yield be achieved. In this field experiment, high NAR under high PPD produced the highest biological yield; however, the harvest index decreased and so lint yield decreased. In contrast, moderate NAR with medium PPD and low NAR with high PPD produced a higher lint yield because of the better regulation of the relationship between boll number and boll weight.

### Effects of PPD and NAR on NUE of cotton

Determination of the optimal PPD and NAR is important to build a more reliable cotton groups, improve utilization efficiency of light[31], and optimize assimilation product distribution to bolls in different canopies[32], ultimately improving the lint yield. Under this field experiment, biological yield and N uptake increased with the increase of PPD and NAR. However, although biological yield and N uptake with high NAR under high PPD were higher than the other treatments, the ratio of assimilation product distributed to reproductive organs decreased, resulting in decreased harvest index and lower economic yield than under medium PPD with moderate NAR and high PPD with low NAR. The average agronomic NUE, physiological NUE and PFPN for the low PPD treatment were better than those for medium and high PPD. Agronomic NUE, physiological NUE, internal NUE, NRE and PFPN declined with the increase of NAR. Low NAR with high PPD produced a comparable yield at a relatively lower NAR, thus the NUE values were superior to those under a moderate NAR with medium PPD.

#### Conclusion

This study has provided new information on the common perception that PPD and NAR affect LAI, biological and economic yield, harvest index, earliness, yield components, agronomic NUE, physiological NUE, internal NUE, NRE and PFPN of cotton under field conditions. We found that the LAI, biological yield, and N uptake of cotton increased with the increase of PPD and NAR, while harvest index and pro-frost yield rate decreased with increasing PPD and NAR. Agronomic NUE, physiological NUE, internal NUE, NRE and PFPN decreased with the increase of NAR. The highest lint yield was obtained at moderate NAR under medium PPD, and a comparable lint yield was achieved with low NAR under high PPD, and high NAR under medium PPD. In contrast, low NAR under high PPD produced superior NRE and PFPN to moderate N under medium PPD. These results indicate that in a medium fertility field in the North China Plain cotton area, maintaining NAR at 112.5–225.0 kg ha^−1^ and increasing PPD to 5.5–6.0 plants m^−2^ could improve both lint yield and NUE.

## Supporting information

S1 Fig Leaf area index(LAI) of cotton at different growth periods in 2013(A) and 2014(B).
Note: D1, D2, D3 indicate planting density at 3.00, 5.25, 7.50 plants m^−2^ respectively, and N0, N1, N2, N3, N4 indicate nitrogen application rate at 0, 112.5, 225.0, 337.5 kg ha^−1^ respectively. Numbers at the same growth stage followed by the same small alphabet are not significantly different at the 5% level.
(DOCX)

S2 Fig N uptake of cotton(A), internal nitrogen use efficiency of cotton(B), partial factor productivity from applied nitrogen of cotton(C), nitrogen recovery efficiency of cotton(D), physiological nitrogen use efficiency of cotton(E) and agronomic nitrogen use efficiency of cotton (F)in 2013 and 2014.
Note: D1, D2, D3 indicate planting density at 3.00, 5.25, 7.50 plants m^−2^ respectively, and N0, N1, N2, N3, N4 indicate nitrogen application rate at 0, 112.5, 225.0, 337.5 kg ha^−1^ respectively. Numbers for the same year followed by the same small alphabet are not significantly different at the 5% level.
(DOCX)

S1 Data Origin data for Fig 1 and Fig 2. (XLS)
