# Supplementary material for: Optimizing nitrogen application rate and plant density for improving cotton yield and nitrogen use efficiency in the North China Plain
Source: PLoS One. 2017 Oct 5;12(10):e0185550. doi: 10.1371/journal.pone.0185550 (PMC5628833; doi:10.1371/journal.pone.0185550)
Supplement: S1 Fig — Leaf area index(LAI) of cotton at different growth periods in 2013(A) and 2014(B). Note: D1, D2, D3 indicate planting density at 3.00, 5.25, 7.50 plants m−2 respectively, and N0, N1, N2, N3, N4 indicate nitrogen application rate at 0, 112.5, 225.0, 337.5 kg ha−1 respectively. Numbers at the same growth stage followed by the same small alphabet are not significantly different at the 5% level. (DOCX) [file pone.0185550.s001.docx]

Fig 1 Leaf area index(LAI) of cotton at different growth periods in 2013(A) and 2014(B)

Note: D1, D2, D3 indicate planting density at 3.00, 5.25, 7.50 plants m^−2^ respectively, and N0, N1, N2, N3, N4 indicate nitrogen application rate at 0, 112.5, 225.0, 337.5 kg ha^−1^ respectively. A, B indicate 2013 and 2014. Numbers at the same growth stage followed by the same small alphabet are not significantly different at the 5% level.
