# Supplementary material for: Optimizing nitrogen application rate and plant density for improving cotton yield and nitrogen use efficiency in the North China Plain
Source: PLoS One. 2017 Oct 5;12(10):e0185550. doi: 10.1371/journal.pone.0185550 (PMC5628833; doi:10.1371/journal.pone.0185550)
Supplement: S2 Fig — N uptake of cotton(A), internal nitrogen use efficiency of cotton(B), partial factor productivity from applied nitrogen of cotton(C), nitrogen recovery efficiency of cotton(D), physiological nitrogen use efficiency of cotton(E) and agronomic nitrogen use efficiency of cotton (F)in 2013 and 2014. Note: D1, D2, D3 indicate planting density at 3.00, 5.25, 7.50 plants m−2 respectively, and N0, N1, N2, N3, N4 indicate nitrogen application rate at 0, 112.5, 225.0, 337.5 kg ha−1 respectively. Numbers for the same year followed by the same small alphabet are not significantly different at the 5% level. (DOCX) [file pone.0185550.s002.docx]

Fig 2. N uptake of cotton(A), internal nitrogen use efficiency of cotton(B), partial factor productivity from applied nitrogen of cotton(C), nitrogen recovery efficiency of cotton(D), physiological nitrogen use efficiency of cotton(E) and agronomic nitrogen use efficiency of cotton(F) in 2013 and 2014

Note: D1, D2, D3 indicate planting density at 3.00, 5.25, 7.50 plants m^−2^ respectively, and N0, N1, N2, N3, N4 indicate nitrogen application rate at 0, 112.5, 225.0, 337.5 kg ha^−1^ respectively. Numbers for the same year followed by the same small alphabet are not significantly different at the 5% level.
